# Epidemiology of Isolated Acromioclavicular Joint Dislocation

**DOI:** 10.1155/2013/171609

**Published:** 2013-01-28

**Authors:** Claudio Chillemi, Vincenzo Franceschini, Luca Dei Giudici, Ambra Alibardi, Francesco Salate Santone, Luis J. Ramos Alday, Marcello Osimani

**Affiliations:** ^1^Department of Orthopaedics and Traumatology, Istituto Chirurgico Ortopedico Traumatologico (I.C.O.T.), Via Faggiana 1668, 04100 Latina, Italy; ^2^Department of Orthopaedics and Traumatology, Sapienza University of Rome, I.C.O.T., Via Faggiana 1668, 04100 Latina, Italy; ^3^Department of Orthopaedics, Marche Polytechnic University, 60121 Ancona, Italy; ^4^Department of Statistics, Value Lab srl, 00187 Rome, Italy; ^5^Department of Orthopaedics and Traumatology, National Institute of Rehabilitation, 14389, Mexico; ^6^Department of Radiological Sciences, Sapienza University of Rome, ICOT, Via Faggiana 1668, 04100 Latina, Italy

## Abstract

*Background*. Acromioclavicular (AC) joint dislocation is a common shoulder problem. However, information about the basic epidemiological features of this condition is scarce. The aim of this study is to analyze the epidemiology of isolated AC dislocation in an urban population. *Materials and Methods*. A retrospective database search was performed to identify all patients with an AC dislocation over a 5-year period. Gender, age, affected side and traumatic mechanism were taken into account. X-rays were reviewed by two of the authors and dislocations were classified according to the Rockwood's criteria. *Results*. A total of 108 patients, with a mean age of 37.5 years were diagnosed with AC dislocation. 105 (97.2%) had an isolated AC dislocation, and 3 (2.8%) were associated with a clavicle fracture. The estimated incidence was 1.8 per 10000 inhabitants per year and the male-female ratio was 8.5 : 1. 50.5% of all dislocations occurred in individuals between the ages of 20 and 39 years. The most common traumatic mechanism was sport injury and the most common type of dislocation was Rockwood type III. *Conclusions*. Age between 20 and 39 years and male sex represent significant demographic risk factors for AC dislocation.

## 1. Introduction

Acromioclavicular (AC) joint dislocation is one of the most common shoulder problems accounting for 9% of all shoulder injuries [1–3], in particular during sport activities which involve contact [4–8]. AC joint dislocations can result from both direct and indirect trauma. Direct trauma is caused by a vertically oriented superior impact on the lateral part of the shoulder, forcing the AC joint in an inferior direction [9]. Indirect trauma generally results from falling on an adducted and outstretched arm causing the humeral head to be driven into the inferior aspect of the acromion and the joint itself [10]. The severity of this condition is directly related to the force of impact. AC joint dislocations range from a simple sprain of the acromion-clavicular and coraco-clavicular ligaments, which are responsible of holding the joint in its physiological position without displacement, to widely displaced injuries with dislocations of the distal third of the clavicle after the delta-trapezial fascia [10]. 

AC dislocations are classified on the basis of the radiographic findings. Different classification systems are available [11, 12], being that of Rockwood et al. [12] the most widely utilized (Table 1).

Despite the large amount of the literature regarding the prognosis and treatment of AC dislocation, there is scarce information regarding the basic epidemiological features of this condition in the general population. Some information can be gathered from studies based on sport practitioners [4–7], but, to our knowledge, there is only one study up to date that assessed the incidence of AC dislocation in a city-like population wherein only 19 cases were reported during a 1-year period [13].

The aim of this study is to analyze the epidemiology of isolated AC dislocation in an urban population during a 5-year period.

## 2. Materials and Methods

A retrospective database search was performed to identify all patients affected with an AC dislocation to the Emergency Department (ED) of a Regional Orthopaedic and Trauma Hospital, serving an area of more than 555.000 inhabitants, between January 1, 2006 and January 1, 2011. Anteroposterior and axillary radiographs of the shoulder were reviewed by 2 of the authors. The interobserver agreement was obtained with a kappa coefficient [14] of 0.85. 

 Gender, age, affected side, and traumatic mechanism were taken into account. AC dislocations were classified according to the Rockwood classification [12]. 

The statistical analysis was performed by a biostatistician (FSS). *χ* ^2^ test was used to analyze differences between genders, for the side of dislocation and traumatic mechanism. The same test was also used to analyze differences between Rockwood's types for mean age and dislocation etiology. The analysis of variance test was performed to determine significant difference in age for Rockwood's type and gender. *P* < 0.05 was considered statistically significant.

## 3. Results

**Figure 1:**
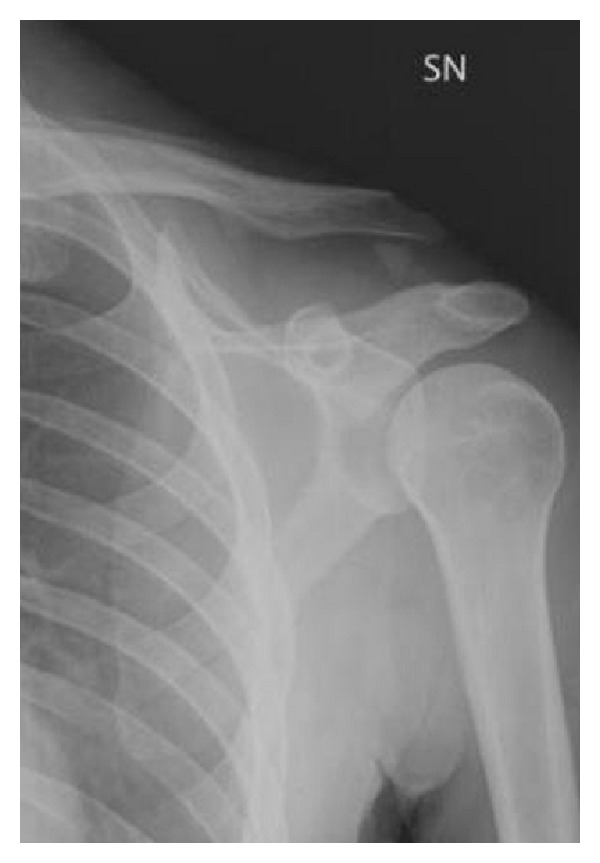
Radiograph (AP view) of a 34-year-old male showing an ACJ dislocation complicated with a fracture of the lateral third of the clavicle.

A total of 108 patients, with a mean age of 37.5 years (range 13–69, SD 13.6), were diagnosed with AC dislocation in the selected 5-year period. Out of these patients, 105 (97.2%) had an isolated AC dislocation, and 3 (2.8%) were associated with a clavicle fracture. In two cases, the fracture involved the lateral third (Figure 1) and in one case the midshaft of the clavicle. As reported in the ED database, the 3 patients were hospitalized and surgically treated with an open reduction and fixation with a hook plate.

The estimated incidence was 1.8 per 10000 inhabitants per year, and the male-female ratio was 8.5 : 1, with 94 (89.5%) male patients and 11 (10.5%) females, which represents a statistically significant difference in gender (*P* = 0.000). In 56 (53.3%) patients, the dislocation was on the right side, while in 49 (46.7%), it was on the left side, which is not a statistically relevant difference (*P* = 0.495). The mean age of females was 36.6 years (range 16–60, SD 15.6), while the mean age of males was 37.6 years (range 13–69, SD 13.5), with no statistically significant difference (*P* = 0.809). 

The demographical data and the distribution of AC dislocation among the population studied are reported in Table 2.

**Figure 2:**
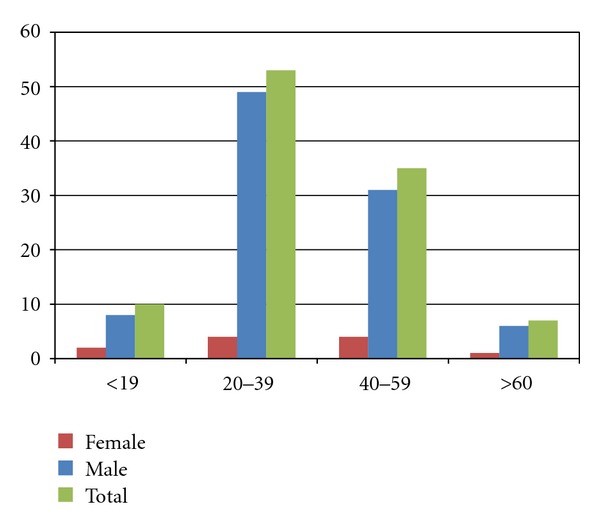
Distribution of patients for age groups and gender. 50.5% of all dislocations (53 cases) occurred in individuals between the ages of 20 and 39 years.

**Figure 3:**
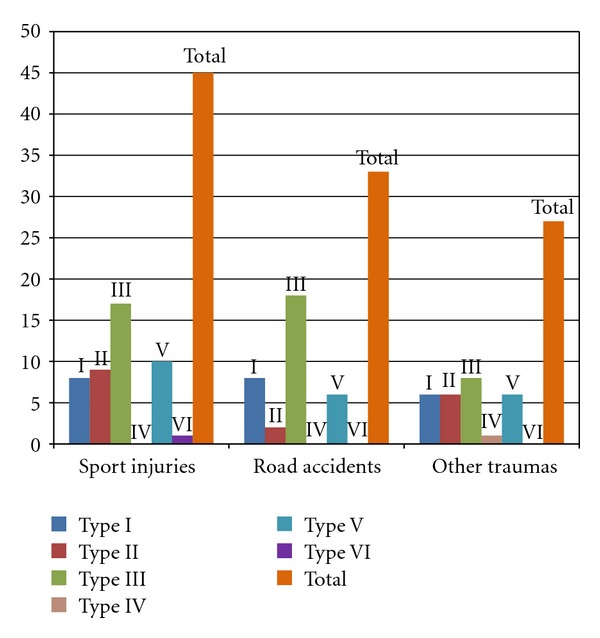
Causes of AC dislocation.

The majority of AC dislocations occurred between the age of 20 and 39 years (Figure 2). The most common traumatic mechanism responsible of AC dislocation was sport injury (cycling 23 cases, soccer 14, basketball 4, and rollerblades 4), accounting for 45 (42.9%) cases, followed by road accident, accounting for 33 (31.4%) cases. Different mechanisms were recognized for the remaining 27 (25.7%) cases: accidental fall (20), work-related injuries (6), and aggression (1) (Figure 3). Additional information regarding the type of treatment was gained from the ED database. 

All patients with type I and type II dislocations were conservatively treated with a sling immobilization for 2 weeks, rest and application of ice. Also, 34 patients (81%) with type III dislocations were conservatively treated with the use of a sling for 4 weeks, while 8 patients (19%) were hospitalized and surgically treated. All patients with type IV, type V, and type VI dislocations were hospitalized and surgically treated.

There was not a significant association between traumatic mechanism and gender (*P* = 0.269), ages over and under 50 years (*P* = 0.058), side of dislocation (*P* = 0.952), and Rockwood's type (*P* = 0.402). Significant associations did not appear even between Rockwood's type and the other observed variables—mean age (*P* = 0.090), sex (*P* = 0.975), and side of dislocation (*P* = 0.522).

## 4. Discussion

It is difficult to provide a comparison with the medical literature available, as most of the epidemiological data concerning AC dislocations can be gathered only through specific papers referring to athletes [4–8]. The only study that assessed the incidence of AC dislocation in a city-like population that we know of is that of Nordqvist and Petersson [13] who reported an incidence of 1.5 per 10000 inhabitants for males and of 0.2 for females. However, it relies on a very small number of AC dislocations (19 cases) over just a 1-year period [13]. This study seems to represent the largest series of AC dislocations referred to an urban population described in the literature.

In most of the cases, AC dislocation is the result of a direct and high-energy impact to the shoulder, which is a frequent occurrence in many sports as well as in road accidents. This explains why injuries to the AC joint are more common in the active population, highly exposed to forceful contacts. In particular, soccer, rugby, and basketball are associated with a higher risk of AC injuries due to tackling or wrong landing after a jump [4–8]. Also, in our study, the majority of dislocations (50.5%) occurred in individuals between 20 and 39 years. The most common traumatic mechanism reported was sport injury, and cycling was found to be responsible for the majority of these lesions. The principal mechanism of lesion regarding cycling is a direct impact to the joint when the arm is adducted or outstretched (putting the AC and coracoclavicular ligaments in a position more susceptible to tears and strains) [15]. The second most common cause of AC dislocation was road accident. In this case, we hypothesize that the tightening effect from the security belts may play a key role in the genesis of the injury, with or without an additional direct trauma to the shoulder. The current study documented a significantly higher incidence rate for males compared to females; this is probably related to differences in life style and hobbies, the men being more inclined to play high-risk activities.

A large amount of literature exists regarding the optimal treatment of AC joint dislocations, which varies depending on the severity of the injury and the soft-tissue involvement. It is well established for types I, II, IV, V, and VI, while the management of type III is still controversial. [1, 16–18]. There is a consensus in the literature that types I and II injuries should be managed nonoperatively. Sling immobilization and symptomatic treatment of pain are usually all that are necessary for these dislocations [16]. Occasionally, pain persists and additional surgical treatment is indicated. Mouhsine et al. [19] found that only 27% of conservatively treated types I and II AC joint dislocations require further surgery at 26 months after injury. If surgery is selected because of the persistence of pain, an excision of the distal part of the clavicle together with a capsular plication and ligament reconstruction should be performed [16]. According to the current evidence, type III dislocations should initially be treated without surgery [1, 12, 16, 20]. In a meta-analysis of 1172 patients with type III dislocations, Phillips et al. [21] reported that 88% of patients who were operatively treated and 87% who were nonoperatively treated had satisfactory outcomes. In addition, complications were more common in the operative group and included the need for further surgery (59% operative versus 6% nonoperative), infection (6% versus 1%), and deformity (3% versus 37%). Larsen et al. [22] also performed a prospective randomized trial of 84 patients, and they found that most patients did as well or better with nonoperative management. Early surgical treatment can be considered for patients with high sports demands, although this is controversial as most of these patients do well with conservative treatment and surgical repair does not restore normal strength to the ligaments of the AC joint [16, 23]. Surgical reconstruction of the ligaments should then be reserved to patients for whom nonoperative treatment has failed [12, 17, 20]. 

Different open [24–26] and arthroscopic [27, 28] surgical techniques are available for reconstructing the injured joint, including repair of the coracoclavicular ligaments with use of sutures, transfer of the coracoacromial ligament to the distal part of the clavicle, augmentation with absorbable and nonabsorbable suture, and coracoclavicular stabilization with screws [17]. Types IV, V, and VI AC dislocations all require early surgical treatment [1, 16]. 

A possible limitation of the study may be seen in the population analyzed. Even if it is definitely more generalized than what is found in other papers [4–8], it still refers to a specific area of Europe, thus reflecting habits and behaviours that might not be found in other countries. In particular, the low incidence of AC dislocation caused by basketball and the absence of cases referred to hockey and rugby might be explained by the low diffusion of these sports in the region. 

## 5. Conclusions

Dislocation of AC joint is not infrequent and should be considered whenever a young adult comes to clinical observation for a direct impact to the shoulder girdle. In the current study, we determined the incidence of pure AC dislocations to be 1.8 per 10000 inhabitants per year. Additionally, we identified an age between 20 and 39 years and male sex as significant demographic risk factors. Even in the urban population, sport activity represents the most common cause of AC dislocation, followed by road accidents. Rockwood's type III is by far the most common presentation.

## Figures and Tables

**Table 1:** The Rockwood classification takes into account not only the acromioclavicular joint, but also the coracoclavicular ligament, the deltoid and trapeziusmuscles, and the direction of dislocation of the clavicle with respect to the acromion. According to this classification, AC dislocations can be divided into 6 types.

Type	AC ligament	AC joint capsule	CC ligament	AC joint displacement	Delta-trapezial fascia
Type I	Sprained	Intact	Intact	None	Intact
Type II	Torn	Disrupted	Intact	50% AC subluxation	Intact
Type III	Torn	Disrupted	Torn	100% AC superior dislocation	Intact
Type IV	Torn	Disrupted	Torn	100% AC posterior dislocation. Posterior displacement of the distal clavicle into or through the trapezius muscle	Disrupted
Type V	Torn	Disrupted	Torn	100–300% AC superior dislocation. Complete detachment of deltoid and trapezius muscle from their clavicular insertion	Disrupted
Type VI	Torn	Disrupted	Torn	100% AC inferior dislocation. Inferior displacement of the distal clavicle into a subacromial or subcoracoid position	Intact

**Table 2:** Demographical data and distribution of AC dislocation among the study population.

	Type I	Type II	Type III	Type IV	Type V	Type VI	Total
No. of patients	22 (21%)	17 (16%)	42 (40%)	1 (1%)	22 (21%)	1 (1%)	105
Left side	11 (50%)	8 (47.1%)	16 (38%)	0	13 (59.1%)	1 (100%)	49
Right side	11 (50%)	9 (52.9%)	26 (62%)	1 (100%)	9 (40.9%)	0	56
Female	3 (13.6%)	2 (11.8%)	4 (9.5%)	0	2 (9.1%)	0	11
Male	19 (86.4%)	15 (88.2%)	38 (90.5%)	1 (100%)	20 (90.9%)	1	94
Mean Age	30.6	38.2	39.0	51.0	40.1	39.00	37.5
